# Where's the Bar? Alcohol and Meaningful Engagement Among Assisted Living Residents With Dementia

**DOI:** 10.1093/geroni/igab046.1117

**Published:** 2021-12-17

**Authors:** Alexis Bender, Candace Kemp, Elisabeth Burgess

**Affiliations:** 1 Emory University, Atlanta, Georgia, United States; 2 Gerontology Institute, Georgia State University, Atlanta, Georgia, United States; 3 Georgia State University, Georgia State University - Atlanta, Georgia, United States

## Abstract

Alcohol use across the life course provides some physical and psychological benefits when used in moderation. As a social model of care, assisted living (AL) communities emphasize autonomy; yet, we do not know how this philosophy extends to drinking. Using ethnographic and interview data from a larger 5-year NIA-funded study in four diverse AL communities designed to identify best practices for the meaningful engagement of AL residents with dementia, we examine how residents, families, and staff interpret residents’ rights about alcohol use and how staff and families facilitate or limit alcohol use of residents with dementia. Findings indicate staff and families frequently rely on a narrative of “watchful oversight” to limit or restrict alcohol consumption while simultaneously affirming the social connection of drinking (e.g., alcohol-free socials). We discuss the implications of our findings for research and practice aimed at promoting meaningful engagement and quality of life among persons with dementia.

